# Crystalline structure, electronic and lattice-dynamics properties of NbTe_2_

**DOI:** 10.1038/s41598-018-35308-4

**Published:** 2018-11-19

**Authors:** Aarón Hernán Barajas-Aguilar, J. C. Irwin, Andrés Manuel Garay-Tapia, Torsten Schwarz, Francisco Paraguay Delgado, P. M. Brodersen, Rajiv Prinja, Nazir Kherani, Sergio J. Jiménez Sandoval

**Affiliations:** 1 Centro de Investigación y de estudios Avanzados del IPN, Unidad Querétaro, Libramiento Norponiente No 2000, Frac., Real de Juriquilla, C.P. 76230 Mexico; 2 Department of Physics, Simon Fraser University, Burnaby, British Columbia V5A 1S6 Canada; 3 Centro de Investigación en Materiales Avanzados, Unidad Monterrey, Apodaca, Nuevo León C.P. 66600 Mexico; 4 Max-Planck-Insitut für Eisenforschung GmbH, Max-Planck-Str. 1, 40237 Düsseldorf, Germany; 5 Centro de Investigación en Materiales Avanzados, Miguel de Cervantes 120, Complejo Industrial Chihuahua, Chihuahua, Chih. C.P. 31136 Mexico; 6 Ontario Centre for Characterization of Advanced Materials, University of Toronto, 200 College Street, Toronto, Ontario M5S 3E5 Canada; 7 The Edward S. Rogers Sr. Department of Electrical and Computer Engineering, University of Toronto, 10 King’s College Road, Toronto, Ontario M5S 3G4 Canada; 8 Department of Material Science and Engineering, University of Toronto, 184 College Street, Toronto, Ontario M5S 3E4 Canada

## Abstract

Layered-structure materials are currently relevant given their quasi-2D nature. Knowledge of their physical properties is currently of major interest. Niobium ditelluride possesses a monoclinic layered-structure with a distortion in the tellurium planes. This structural complexity has hindered the determination of its fundamental physical properties. In this work, NbTe_2_ crystals were used to elucidate its structural, compositional, electronic and vibrational properties. These findings have been compared with calculations based on density functional theory. The chemical composition and elemental distribution at the nanoscale were obtained through atom probe tomography. Ultraviolet photoelectron spectroscopy allowed the first determination of the work function of NbTe_2_. Its high value, 5.32 eV, and chemical stability allow foreseeing applications such as contact in optoelectronics. Raman spectra were obtained using different excitation laser lines: 488, 633, and 785 nm. The vibrational frequencies were in agreement with those determined through density functional theory. It was possible to detect a theoretically-predicted, low-frequency, low-intensity Raman active mode not previously observed. The dispersion curves and electronic band structure were calculated, along with their corresponding density of states. The electrical properties, as well as a pseudo-gap in the density of states around the Fermi energy are characteristics proper of a semi metal.

## Introduction

Transition metal dichalcogenides (TMD’s) are quasi-2D materials with layered structures consisting of stacks of chalcogenide-transition metal-chalcogenide (M-X-M) unit layers bound by weak van-der-Waals-type forces^1^. This weak interlayer attraction allows obtaining layers of a few-monolayer thicknesses by exfoliating the surface of the bulk material^2^. This property makes these materials both technological and fundamentally relevant. In the case of niobium ditelluride, it has been determined that it is a TMD with metallic-type behavior at temperatures higher than 0.5 K, acquiring superconductor properties below this temperature^3^. Niobium ditelluride has a complex crystalline structure consisting of buckled Te-Nb-Te layers, alternating with van-der-Waals gaps. This arrangement may be considered as a *distorted* 1T structure. In the ideal (undistorted) 1T structure, the metal atoms are octahedrally coordinated to surrounding chalcogen atoms. The vibrational properties of NbTe_2_ were studied in the past by Erdogan and Kirby^4^. From their measurements in NbTe_2_ crystals, eleven Raman active modes with frequencies between 56 and 254 cm^−1^ were determined. The theoretical analysis of these results was carried out by considering a simple nearest-neighbor force constant model for a single layer of an undistorted 1T- CdI_2_ structure (*i.e*. considering undistorted, flat, Te-Nb-Te layers) utilizing the force constants of TaS_2_. The effects on the proposed phonon dispersion curves, induced by the actual distortion of the layers were discussed in terms of symmetry considerations. This approach, however, led to mistakenly predicting 51 optical phonon modes. More recently, C. Batagglia *et al*.^5^ carried out Fermi surface studies in order to investigate the origin of the distortion in the monoclinic structure of NbTe_2_. These studies included density functional theory (DFT) calculations of the electronic band structure and phonon dispersion curves of NbTe_2_ using the related, undistorted 1T structure. Since in these calculations the actual structure of NbTe_2_ was not considered, imaginary frequencies were obtained for acoustic phonons along three high-symmetry directions of the Brillouin zone. Additionally, it may be noted that group theory predicts two Raman active modes for the undistorted 1T structure, while consideration of the real distorted monoclinic structure in the decomposition into irreducible representations of NbTe_2_ leads to twelve vibrational modes with Raman activity (*vide infra*).

In this paper, we present first-principle DFT calculations of the electronic and lattice dynamics properties for the actual *distorted* monoclinic structure of NbTe_2_. The electronic band structure and density of states, as well as the one-phonon dispersion curves and density of states are presented. The calculated frequencies of the Raman active modes were in good agreement with high-resolution, high-throughput, inelastic light scattering experiments carried out on structural and chemically characterized NbTe_2_ crystals. The characterization included X-ray diffraction, scanning and transmission electron microscopies, as well as atom probe tomography measurements. From Hall effect and ultra-violet photoelectron spectroscopy experiments, electrical properties such as free carrier concentration, mobility and work function were determined. These properties have not been previously reported for niobium ditelluride. It is pointed out that the high value of the work function of NbTe_2_ determined in this work, namely 5.32 eV, can be of interest for optoelectronics applications, where high-work-function conducting materials are rather scarce and valued.

## Results

### Experimental structural and compositional properties

**Figure 1:**
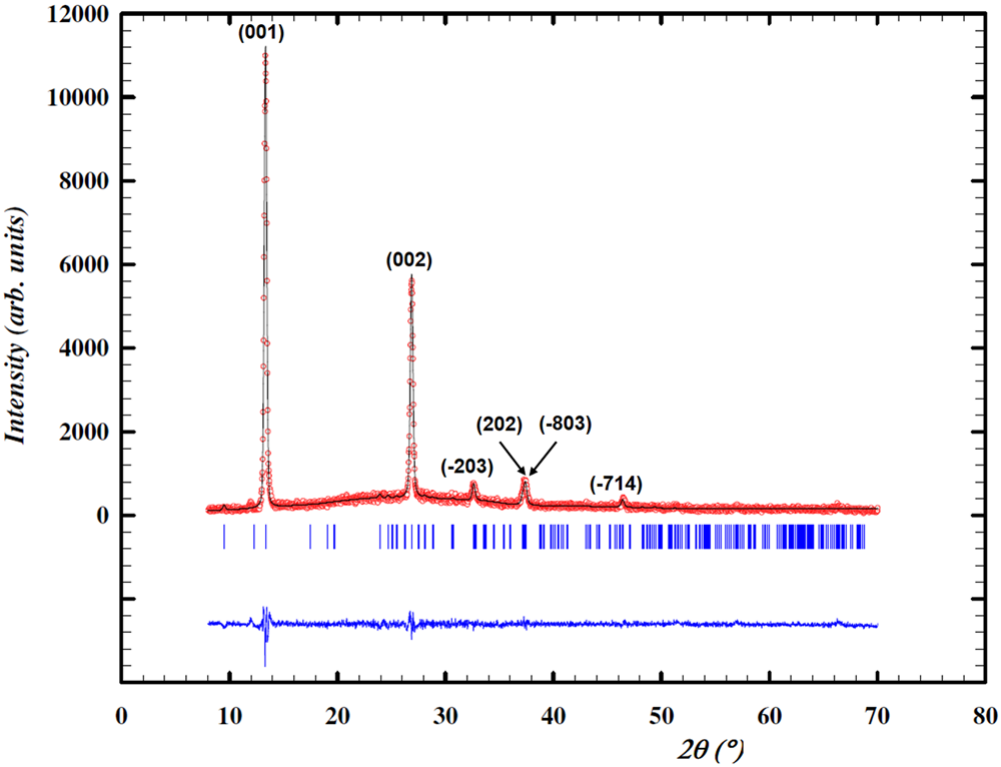
X-ray diffraction pattern of the NbTe_2_ crystal and whole pattern fitting carried out by the Rietveld method. Circles correspond to the experimental data, while the fitted pattern is indicated by the solid line. The difference between the experimental data and the fit is described by the bottom graph.

**Figure 2:**
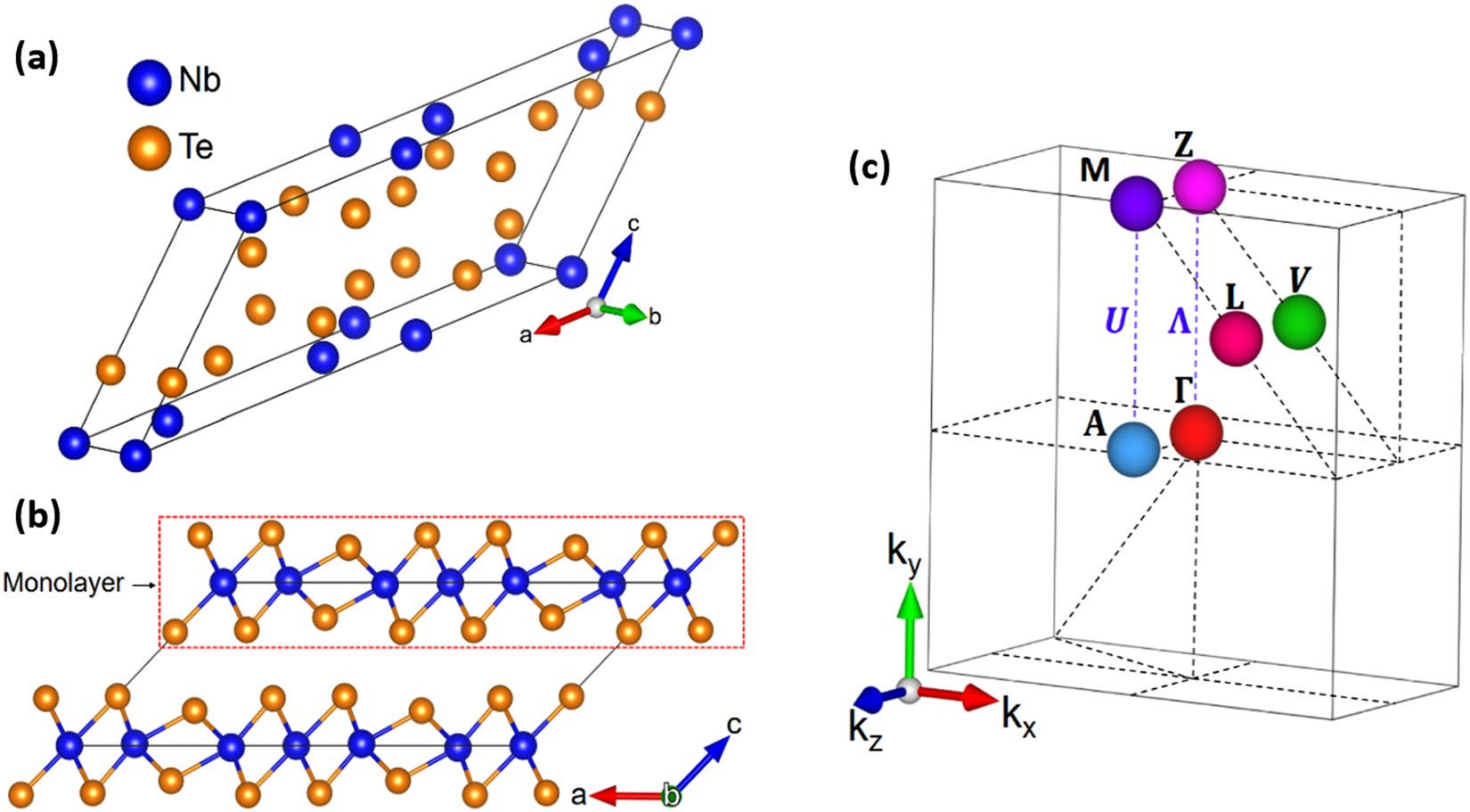
(**a**) NbTe_2_ conventional monoclinic cell; (**b**) stacking sequence of the monolayers in NbTe_2_. (**c**) First Brillouin zone and its high symmetry points and directions. The colors of the high-symmetry points in the Brillouin zone are in correspondence with the vertical lines in Figs 12 (electronic band structure) and 13 (phonon dispersion curves).

X-ray diffraction was used to identify the crystalline phase of the NbTe_2_ samples. From the obtained diffractogram (Fig. 1) and the data in ref.^6^ it was determined that the crystalline structure is monoclinic, described by the symmetry space group 12 (C2/m). The corresponding unit cell is shown in Fig. 2a. Given the layered nature of NbTe_2_ and a preferred growth orientation, the most intense reflections in the diffraction pattern correspond to the [00*c*] direction, Fig. 1. The stacking sequence of the single layers for this material is shown in Fig. 2b. As a consequence of the structural distortion in NbTe_2_, the buckled surface of the Te planes and the non-uniform Nb-Nb distances can be observed.

**Figure 3:**
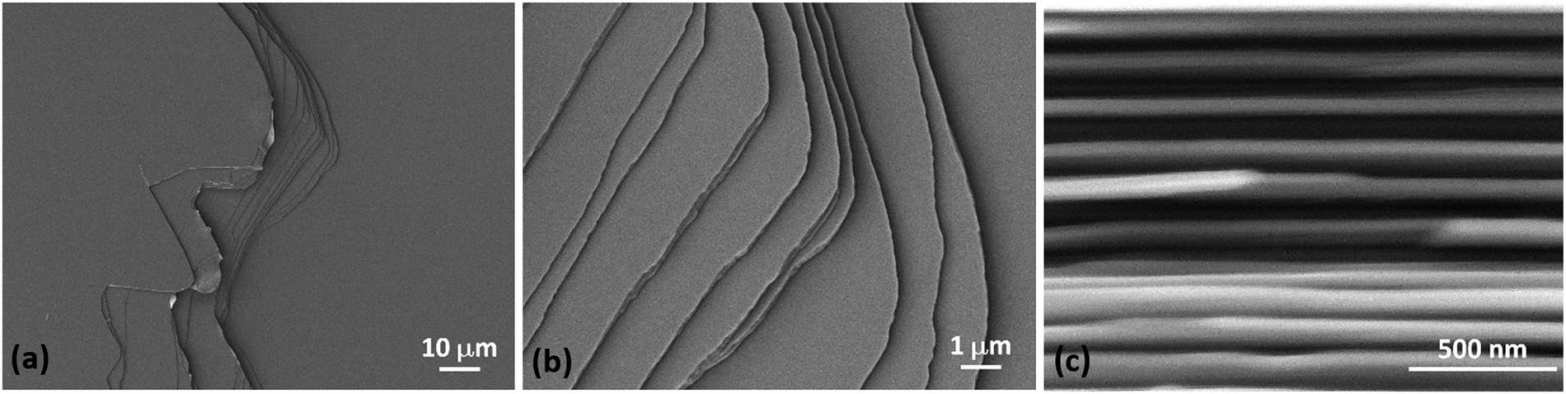
SEM images of the (**a**,**b**) upper and (**c**) cross-sectional views of exfoliated NbTe_2_ crystals at different magnifications.

The upper surface and lateral cross-section morphologies were analyzed by SEM on freshly exfoliated surfaces, Fig. 3. The quasi-2D nature of the stacked layers is evident in the upper views, Fig. 3a,b, as well as in the cross-sectional micrograph, Fig. 3c. The images show homogeneous and flat surfaces at the scales indicated in Fig. 3a,b except when the edges of several layers are exposed, so that a terrace-like structure is apparent.

Although there is good agreement in the position of the experimental and the reported diffraction peaks, the (−203) and (−714) experimental reflections present a slight displacement to smaller angles. This kind of displacements can be caused by a difference between the sample’s lattice parameters and/or angles, and the reported values in the powder diffraction file (PDF). According to the PDF 21-0605, the lattice parameters for NbTe_2_ are: *a* = 19.39 Å, *b* = 3.64 Å and *c* = 9.38 Å, with the angles α = 90°, β = 134.58° and *γ* = 90°. In order to quantify the difference, a whole pattern fitting was made by the Rietveld method^7^ using the software FullProf^8^, in which the position, width and intensity of each reflection was adjusted. The fitted pattern is shown with the solid line in Fig. 1. From this analysis it was determined that the angles and lattice parameters of the NbTe_2_ crystals used in this work were: *a* = 20.98 Å, *b* = 3.66 Å and *c* = 9.65 Å, with α = 90°, β = 136.6°, *γ* = 90° respectively.

**Figure 4:**
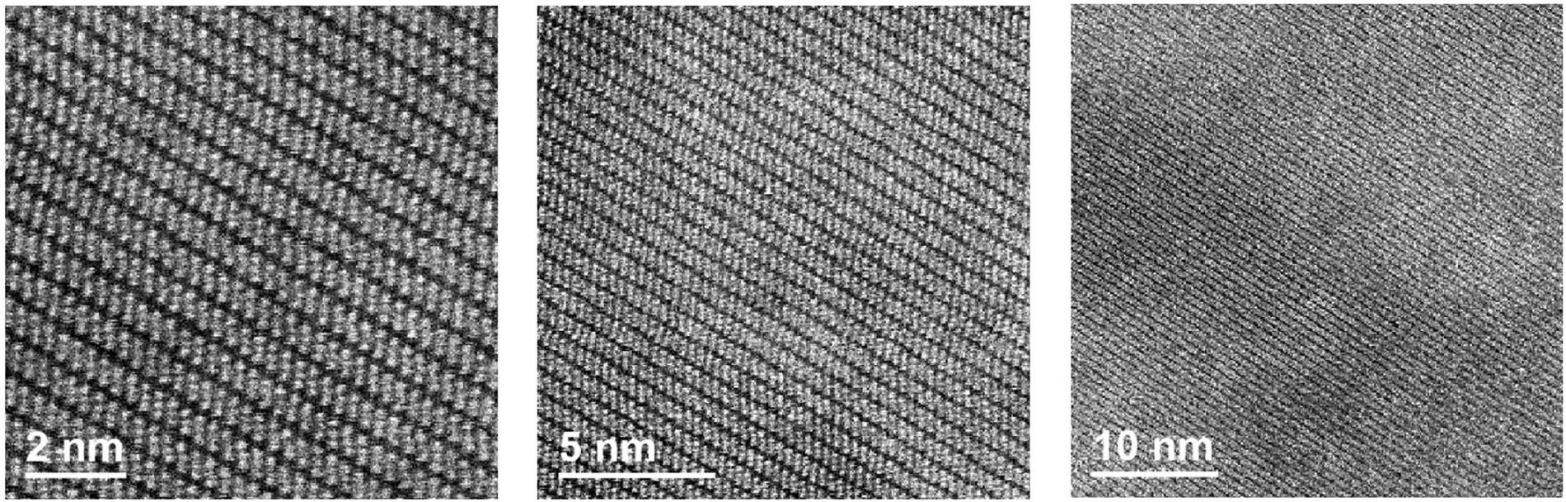
HRTEM cross section images of NbTe_2_ at different magnifications. Atomic resolution may be observed in the first two images. The darker lines correspond to the van der Waals regions separating the Te-Nb-Te layers.

**Figure 5:**
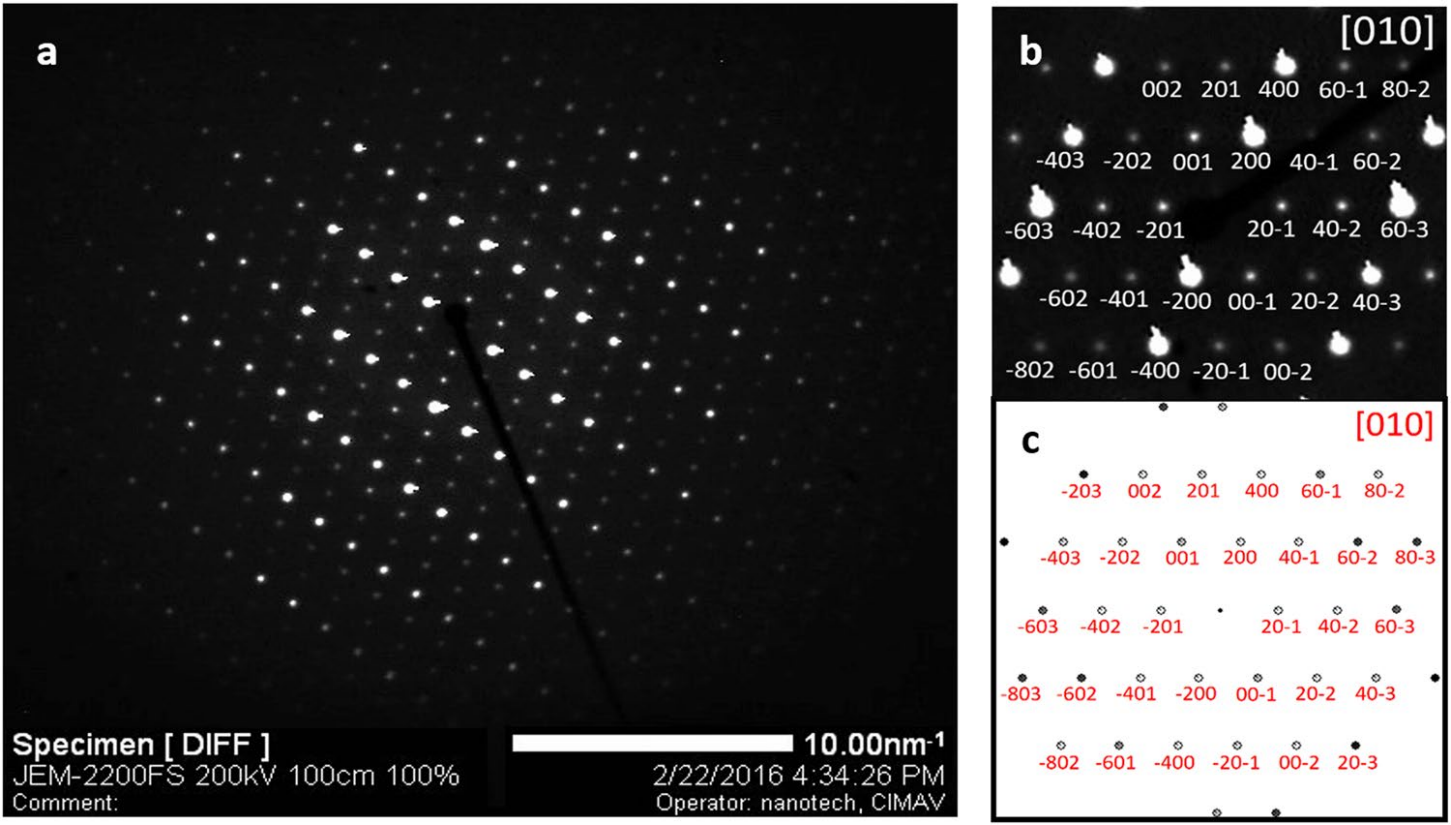
(**a**) Non- indexed, (**b**) indexed and (**c**) simulated selected area diffraction patterns (SADP) of NbTe_2_. The zone axis is the [010] direction.

**Figure 6:**
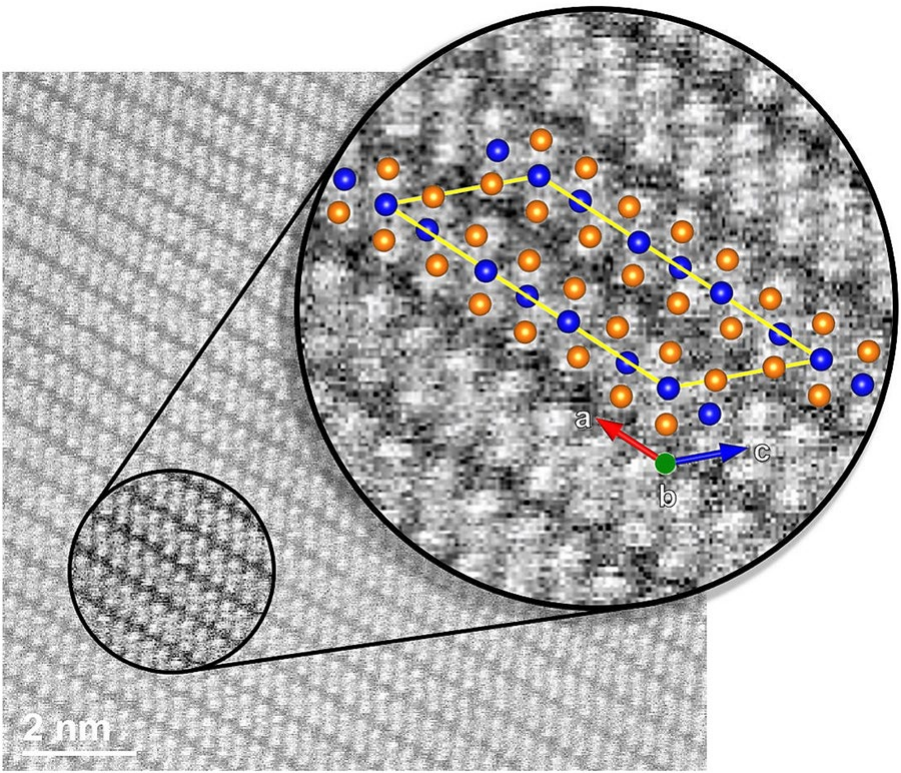
Superposition of a HRTEM image and the *a-c* plane view of the NbTe_2_ unit cell obtained from the Rietveld refining of the X-ray diffraction patterns. A good match in the atomic positions was obtained.

To further investigate the structural properties of niobium ditelluride, cross-sectional HRTEM images were obtained at different magnifications, Fig. 4. The atomic positions in the layered structure are readily observable as well as the high crystalline quality and homogeneity of the sample. The van der Waals gaps appear as slightly darker stripes in all images of Fig. 4. It was possible to obtain selected area diffraction patterns (SADP), as the one shown in Fig. 5a. The pattern exhibits well defined bright diffraction spots, immersed in a dark background. The good contrast between white spots and dark background is indicative of high crystalline quality. The lattice parameters and unit cell angles determined from the Rietveld analysis of the X-ray parameters were employed to obtain the indexation and simulation of the SADP (Fig. 5b,c, respectively). This was accomplished with the help of the software STEM_CELL^9,10^. From this analysis it was found that the zone axis of the SADP was the [010] direction. It is worth pointing out the good agreement between the calculated angles and lattice parameters obtained from the Rietveld method with those measured directly in the HRTEM images. Indeed, a good match in the atomic positions is obtained when the simulated crystalline structure and an HRTEM image are overlapped, as shown in Fig. 6.

**Figure 7:**
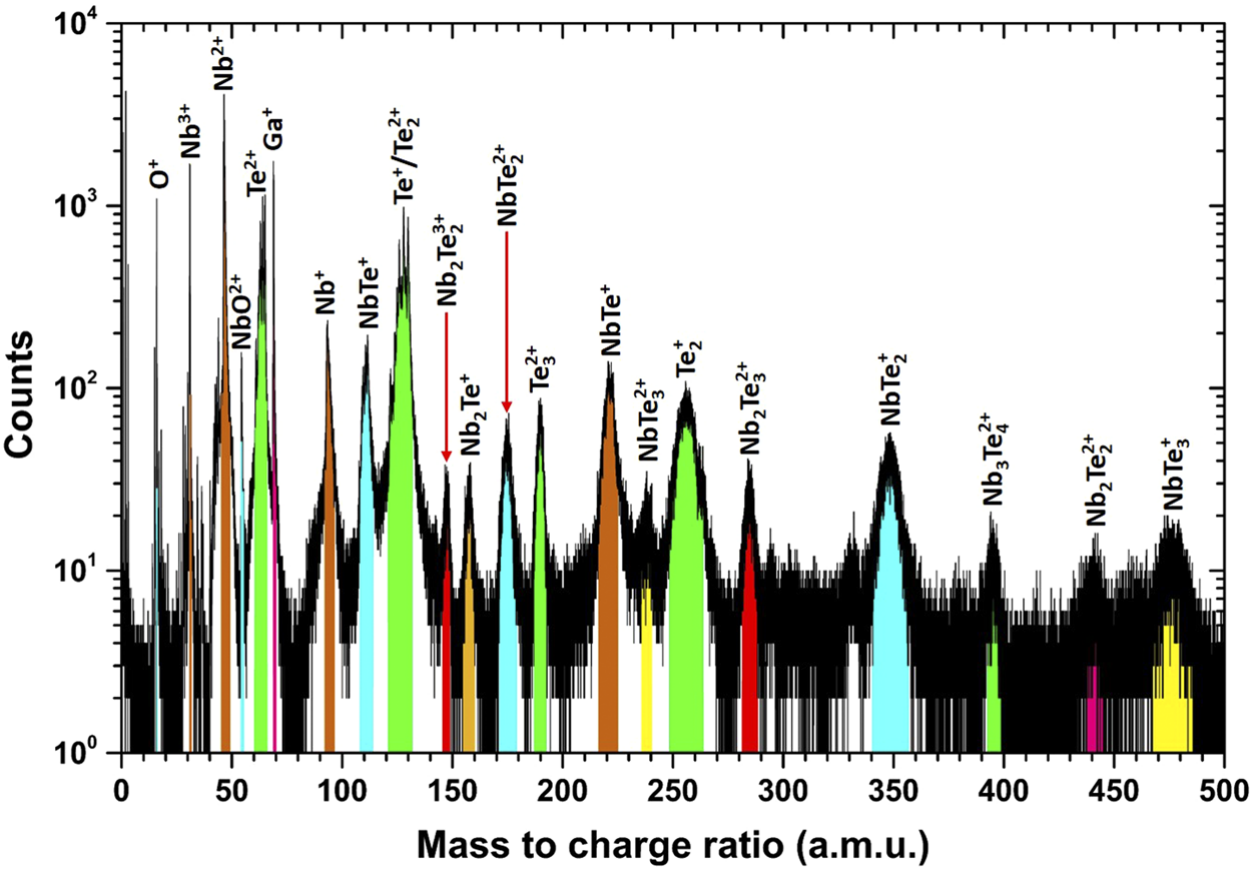
APT mass spectrum of the NbTe_2_ crystal.

**Figure 8:**
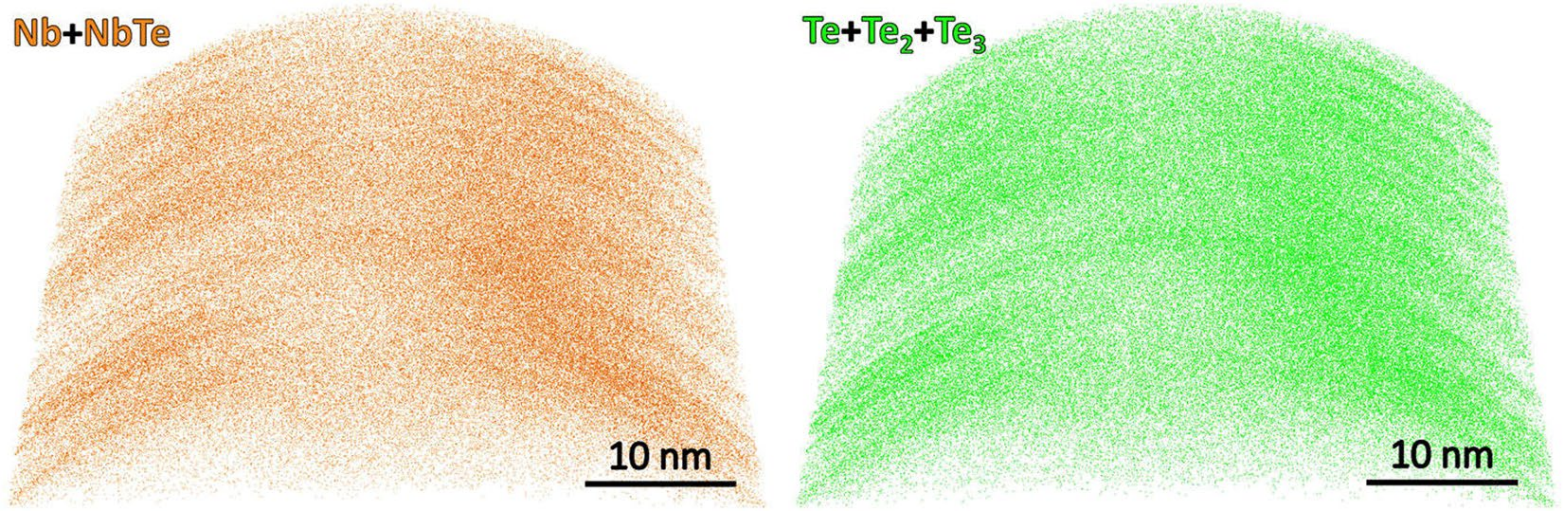
Elemental distribution at the nanoscale obtained from the atom probe tomography measurements for the indicated species.

The elemental composition was determined from atom probe tomography experiments. From these measurements, it was found that the NbTe_2_ crystals consisted of: Nb = ~38 at.%, Te = ~61 at.% and O = ~1 at.%. This composition differs from the ideal ratio of [Te]/[Nb] = 2; instead we have [Te]/[Nb] = 1.6. The Te deficiency might be caused by a preferential loss of Te during the APT analysis, which is a known problem for semiconductor compounds^11,12^. The corresponding mass spectrum is shown in Fig. 7, where all detected ionic species can be observed. A large fraction of the detected ions corresponds to complex, heavy molecular Nb-Te species. The existence of such complex ions might be to some extend due to the weak van-der-Waals bonding. Nb-O species were detected only in the form of molecular NbO^2+^ ions. The origin of the O related mass peaks, which sum up to ~1 at.% O in the APT dataset, is not clear. One origin is oxidation of the surface of the specimen and another due to the residual gases in the analysis chamber. A small fraction of the O may come from the specimen itself replacing Te. Figure 8 shows the 3D reconstruction of the sample at the nanoscale for Nb and Te species. There is good homogeneity in the Nb and Te distributions, with the observation that regions with slightly higher Nb density match with Te regions with the same characteristic. Those high-density regions are due to a rather interruptive field-evaporation behavior, which might be caused to some extend by the weak van-der-Waals gaps in the material.

### First principle calculations: NbTe_2_ crystalline structure

Based on the crystallographic information file (CIF) of NbTe_2_, a structural analysis was carried out using the VASP code. The lattice parameters calculated for the relaxed structure in VASP were: *a* = 19.59 Å, *b* = 3.66 Å and *c* = 9.64 Å, with unit-cell the angles of: and α = 90°, β = 134.2°, *γ* = 90°. A comparison between the lattice parameters obtained in this work (Rietveld refinement and VASP optimization) and those reported in the PDF file is presented in Table 1. In general, there is good agreement among the obtained values. The parameters obtained from the Rietveld refinement, though, tend to be slightly larger than the rest.

**Table 1:** Lattice parameters of NbTe_2_ from the PDF file No. 21-0605, Rietveld refinement of the X-ray diffraction pattern (this work), and from the VASP-code optimization (this work).

	*a* (Å)	*b* (Å)	*c* (Å)	*α*	*β*	*γ*
PDF No. 21-0605	19.39	3.64	9.38	90°	134.58°	90°
Rietveld Refinement	20.98	3.66	9.65	90°	136.6°	90°
VASP	19.59	3.66	9.64	90°	134.2°	90°

Cohesive energy (or binding energy) and heat of formation were also calculated by DFT. In the case of TMD’s with chemical formula MX_2_ the cohesive energy per formula-unit is defined as:

$${E}_{B}(M{X}_{2})={E}_{MX2}-{E}_{Matom}\,-\,2{E}_{Xatom},$$

where E_MX2_ is the total energy of the MX_2_ formula-unit in the bulk material, E_M atom_ and E_X atom_ are the energies of the corresponding M and X isolated atoms, *i.e*. Nb and Te, respectively. It was found that E_B_(MX_2_) = −13.22 eV/formula-unit or −1273.8 KJ/mol. On the other hand, the energy of formation can be calculated as^13^:

$${\Delta }{E}_{f}(M{X}_{2})={E}_{MX2}\,-\,{E}_{Mst}\,-\,2{E}_{Xst},$$

where *E*_*MX2*_ is the total energy of the MX_2_ formula-unit in the bulk material, *E*_*M st*_ and *E*_*X st*_ are the energies of the corresponding M (Nb) and X (Te) atoms at their stable structures^14,15^. Since all the calculated energies correspond to relaxed structures, the effect of pressure is neglected, and the energy of formation is approximated to the enthalpy of formation; that is^16^,

$${\Delta }{E}_{f}(M{X}_{2}) \sim {\Delta }{H}_{f}(M{X}_{2}).$$

**Figure 9:**
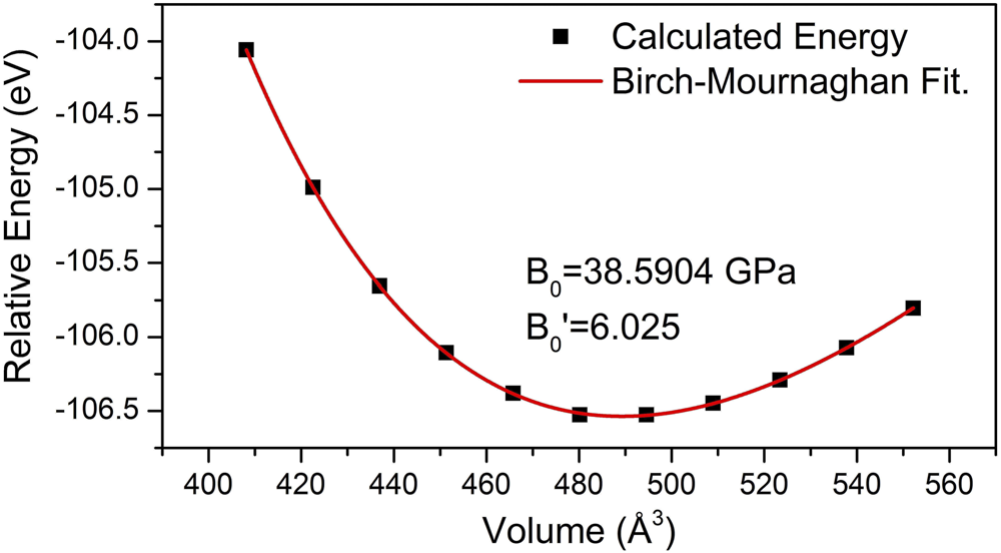
Calculated relative energy as a function of the unit cell volume.

In this case, since the energies are computed at 0 K, the term *heat of formation* is more accurate because the definition of *enthalpy of formation* implies the material´s energy at 298 K^15^. Thus, ΔH_f_ (NbTe_2_) = −1.38 eV/formula-unit or −132.85 KJ/mol. This negative value is a measure of the structure stability^17^. An analysis of the energy *vs*. volume curve, Fig. 9, was made to calculate the bulk modulus (B_0_) of the material. For solids, B_0_ is the ratio of the applied hydrostatic pressure to the corresponding change in volume^18^. Volume compressions and expansions were performed to calculate the energy of the material under hydrostatic strain and stress. The maximum deformation of the volume was 15% for both compression and expansion. Then, with the purpose of computing B_0,_ a least squares fitting was carried out using the Birch-Mournaghan equation^17,19^. The calculated bulk modulus and its derivative were in this case B_0_ = 38.5904 GPa and B_0_’ = 6.025, respectively^16^.

### Electronic Properties

**Figure 10:**
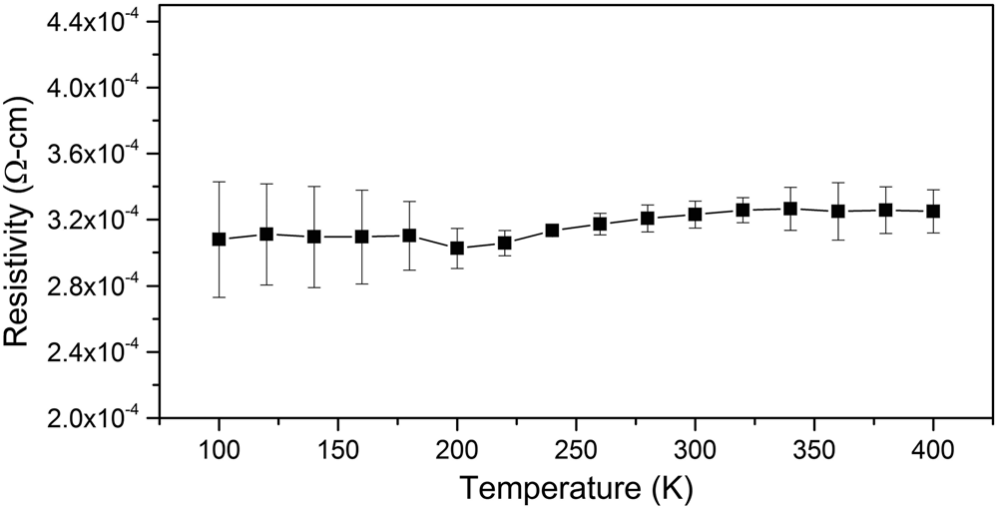
Resistivity as a function of temperature for NbTe_2_. Contacts were made to a freshly exfoliated surface of the crystal.

Although some work on the electronic properties of NbTe_2_ has been carried out, there are still some important properties of the material that remain unknown to date. For example, there are no reports on relevant electrical parameters such as carrier concentration, mobility or work function. Here, we report the results of Hall effect measurements performed at room temperature on square (5 × 5 mm^2^) NbTe_2_ crystals using the Van der Pauw method. We found a carrier concentration of *n* = 5.46 × 10^21^ cm^−3^ with a mobility of μ = 6.9 cm^2^/Vs, resulting in a resistivity of ρ = 1.74 × 10^−4^ Ω·cm. This value agrees well with previous measurements of ρ = 1.8 × 10^−4^ Ω·cm and ρ = 2.6 × 10^−4^ Ω·cm (both at room temperature) reported in ref.^3^ and^20^, respectively. Additionally, using the Van der Pauw method, the resistivity was measured as a function of temperature (100–400 K) in a different system. That is the reason for the slight difference between the room temperature value of ρ given above and that in Fig. 10, which shows the whole temperature dependency. The resistivity of each sample was measured four times in the whole temperature range. The results reported in Fig. 10, correspond to the average resistivity from these measurements with the corresponding standard deviation as error bars. It is noticeable in this figure that there is no significant variation of the resistivity in the investigated temperature range. A slight drop is appreciable as the temperature of the sample decreases; nevertheless, the resistivity remains basically constant. Similar results were obtained by Nagata *et al*.^3^, as they report resistivity changes lower than an order of magnitude when the temperature of the sample changed from 280 to 0.7 K. Brixner^20^ found resistivities of ρ = 2.6 × 10^−4^ Ω·cm at 298 K and ρ = 7.7 × 10^−4^ Ω·cm at 77 K. Along the same line of results, Battaglia *et al*.^5^ presented angle-resolved photoelectron spectroscopy spectra taken at room temperature, finding that the same spectra were obtained at temperatures lower than 20 K, which is an indication that the topology of the NbTe_2_ electronic band structure is basically unaltered before significant temperature changes. Coincidentally, a similar behavior is observed for the magnetic susceptibility, which is null since 725 K up to ~0.5 K, when the material reaches its critical temperature and becomes superconductor^3,21^.

**Figure 11:**
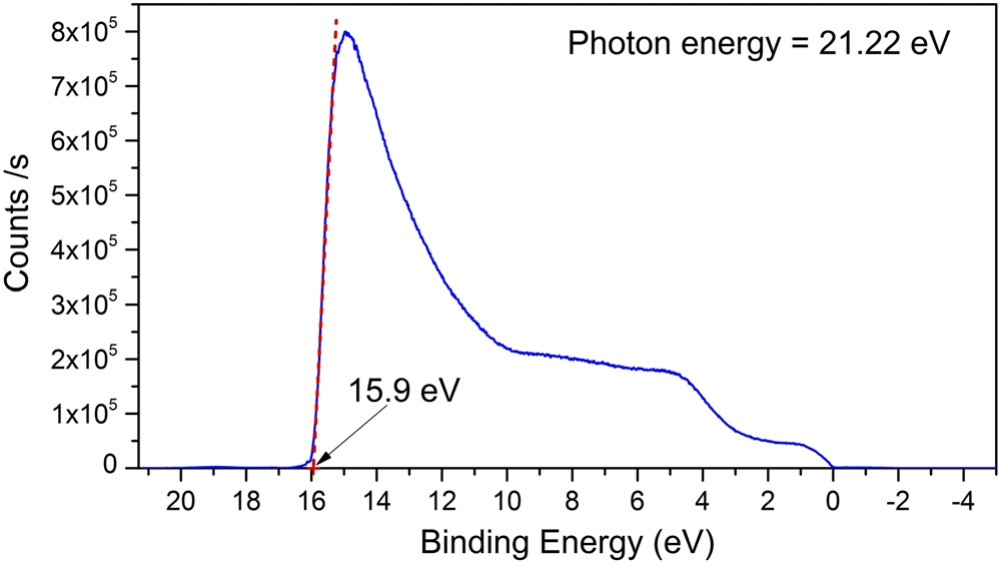
Ultraviolet photoelectron spectroscopy data for NbTe_2_. The arrow indicates the value of *BE*_*max*_ that was used to determine the work function for niobium ditelluride.

Ultraviolet photoelectron spectroscopy (UPS), a technique whose foundations rely on the photoelectric effect, was used to determine the work function of NbTe_2_. The light source was a monochromatic lamp of He I (*hν* = 21.22 eV). Figure 11 shows the curve of emitted photoelectrons as a function of the binding energy. The analysis of the data is based on the fact that the Fermi level is set at 0 eV. Then, the work function is the difference between the incident photon energy and the maximum energy of the emitted photoelectrons, that is *ϕ* = *hν* − *BE*_*max*_. From Fig. 11, it may be observed that *BE*_*max*_ = 15.9 eV. Thus, the work function for NbTe_2_ is *ϕ* = 5.32 eV. This work function is close to that of gold that has been reported with values ranging from 5.2 to 5.3 eV^22^, one of the largest among metals. Large work functions are particularly valuable in cases where electrical contacts to *p*-type semiconductors are necessary. It must be mentioned, however, that the resistivity of gold is about two orders of magnitude lower than that of NbTe_2_. The niobium ditelluride crystals used in this work were stored in ambient conditions for more than one year. It was found that the electrical properties of freshly cleaved surfaces were basically unchanged after long storage periods. Moreover, the data of Fig. 10 show that the resistivity remains steady before significant temperature variations. These characteristics make NbTe_2_ an interesting material not only for applications, such as optoelectronic devices fabrication, but also from the fundamental properties point of view. The fact that the resistivity is unaltered for temperatures between 100 and 400 K is indicative that the electron-phonon interaction is not significant for the charge transport process in that temperature range.

**Figure 12:**
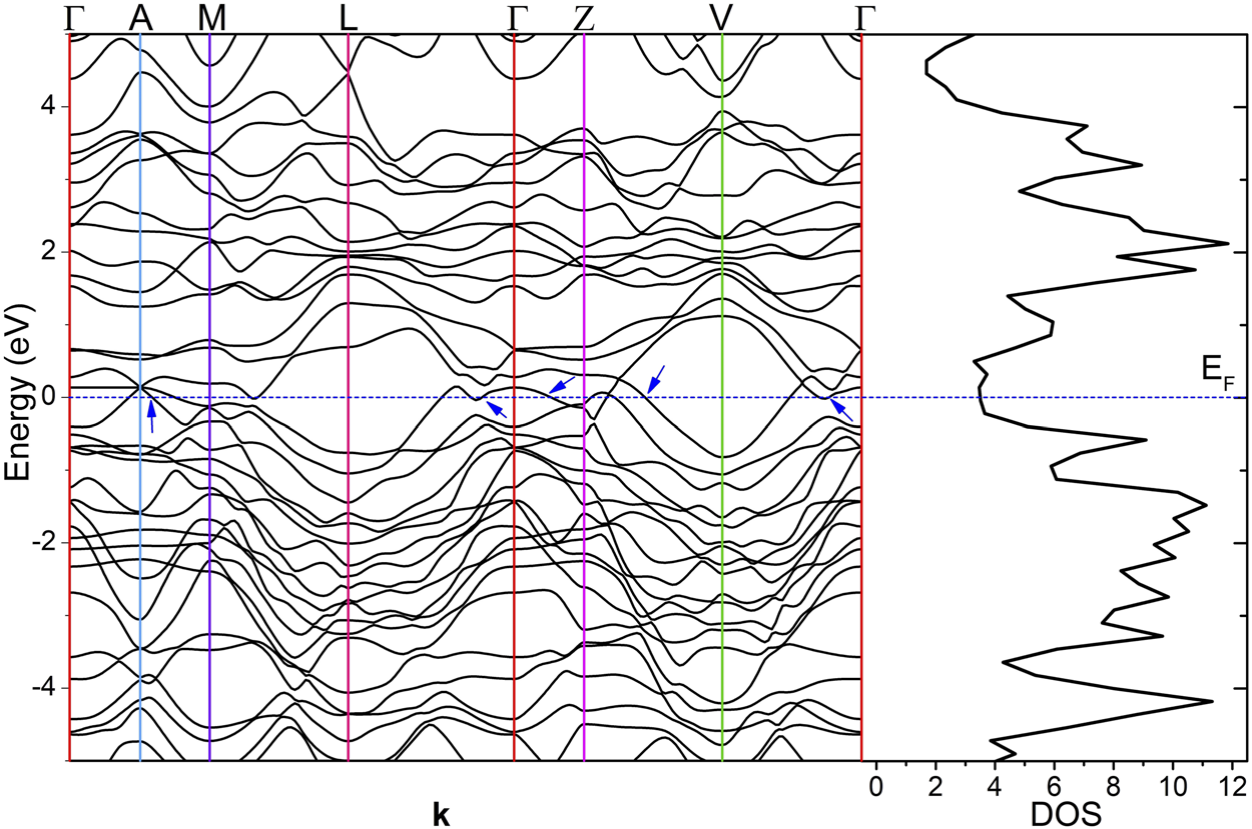
Electronic band structure and density of states of NbTe_2_. The arrows indicate examples of band crossings around E_F_ that produce partial fillings of valence and conduction bands. The high symmetry directions and points of the Brillouin zone are shown in Fig. 2c. As a guide, the colors of the high symmetry points of the Brillouin zone in Fig. 2c and the color of the corresponding vertical lines in Fig. 12 are in correspondence.

One of the most important aspect of materials properties is the electronic band structure and its corresponding density of states (DOS). Battaglia *et al*.^5^ reported DFT calculations of the band structure for trigonal NbTe_2_, *i.e*. for the approximate, ideal, 1 T phase, finding metallic behavior. As mentioned above, the real structure of NbTe_2_ differs from the 1T phase, presenting buckled Te planes that change the symmetry of the crystal from trigonal to monoclinic. Kumar *et al*.^23^ used DFT to calculate the band structure and DOS for a hypothetical hexagonal phase of NbTe_2_ (2H polytype), finding metallic behavior as well. Although the calculations were carried out for comparison purposes with different niobium dichalcogenides with hexagonal structure, the 2H phase has never been observed for NbTe_2_. In this work it is reported the band structure and DOS of niobium ditelluride considering the real structure of the material (*i.e*. monoclinic symmetry with buckled Te planes) using first-principle DFT calculations. Figure 12 shows the electronic band structure of NbTe_2_ along the high-symmetry directions of the Brillouin zone, which is depicted in Fig. 2c. The Fermi level (E_F_) is indicated by the dotted line. It crosses bands along several directions of the Brillouin zone as it occurs in metallic systems (examples are indicated by the arrows in Fig. 12). However, a good number of bands cross from above and below E_F_ to the opposite side around the Fermi level, leaving partially filled valence and conduction bands. This observation, the resistivity of the order of 10^−4^ Ω·cm, and the fact that the DOS around the Fermi energy is close to a minimum, *i.e*. forming a pseudogap (distinguished by a relatively small number of electronic states), are distinctive properties of a semimetal. As comparison, the resistivity of metallic materials is typically within the range of 10^−5^–10^−6^ Ω·cm, with conduction electron densities greater than 10^22^ cm^−3^. Common semimetals have resistivities of the order of 10^−4^ Ω·cm, with free-carrier densities between 10^17^ and 10^20^ cm^−3^ ^24^.

### Lattice dynamics

**Figure 13:**
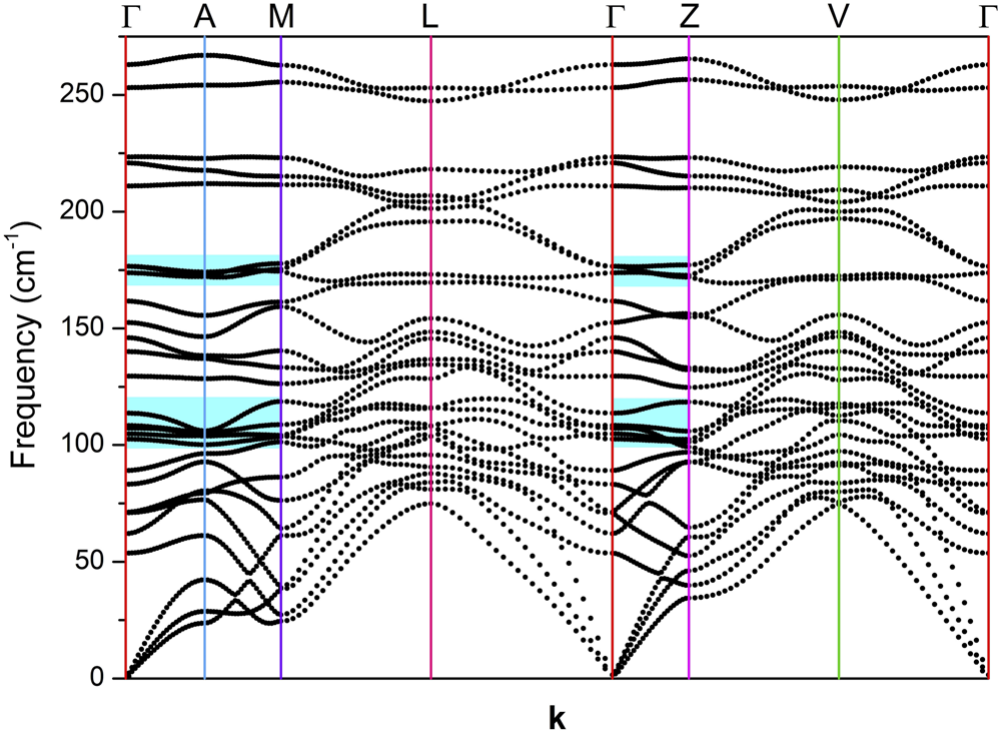
Phonon dispersion curves of NbTe_2_. The high symmetry directions and points of the Brillouin zone are shown in Fig. 2c. As a guide, the colors of the high symmetry points of the Brillouin zone in Fig. 2c and the color of the corresponding vertical lines in this figure are in correspondence. The shadowed regions correspond to phonon energies with low dispersion (*i.e*. high density of states) along the high-symmetry directions Z-Γ-A-M. The energy separation of these zones is in agreement with the energy of the X-mode (60 cm^−1^, see below) in a phonon-difference process. At the zone center (Γ), this process corresponds to the difference B_g_^4^ (169.7 cm^−1^) − B_g_^3^ (109.8 cm^−1^), where the quoted frequencies are experimental.

**Figure 14:**
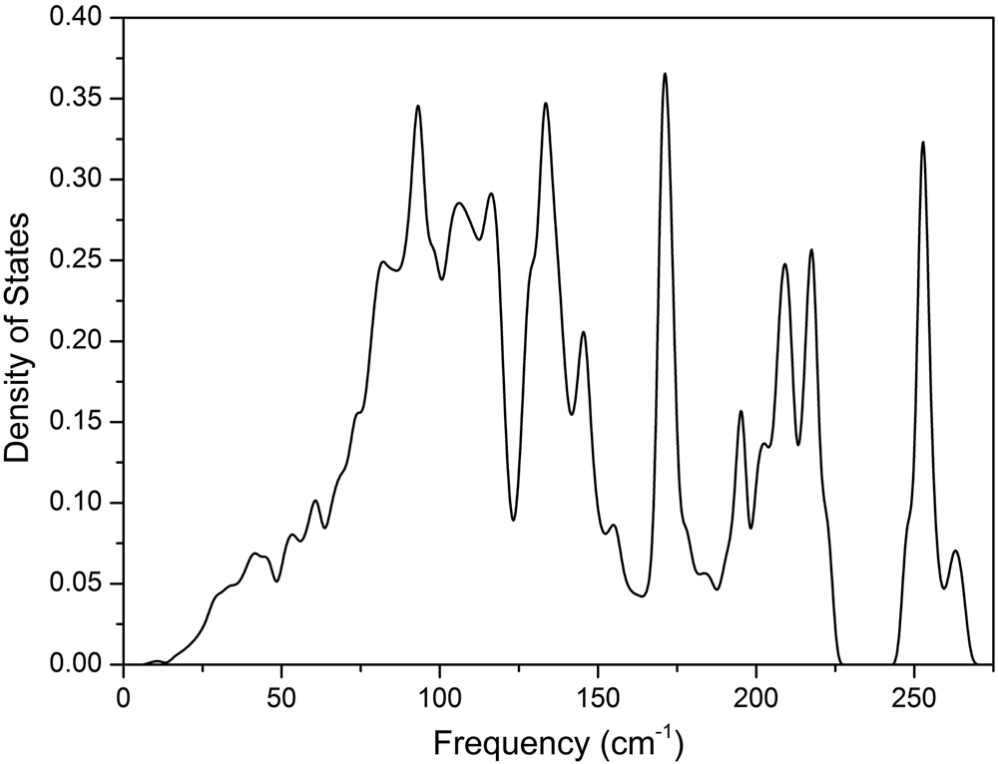
Phonon density of states of NbTe_2_.

The lattice dynamics of a material is related to important physical properties such as electronic, thermodynamical and, in general, the material´s energetics. This occurs due to several mechanisms that involve interactions between other elementary excitations and phonons, which highlights the relevance of determining the lattice dynamics of a material in order to gain a good understanding of its physical properties. To date, the vibrational properties of NbTe_2_ have not been determined for the actual monoclinic structure. Here we present the results of DFT calculations of the phonon dispersion curves (PDC) and the corresponding DOS considering the real crystalline structure of NbTe_2_. In addition, the experimental frequencies determined by Raman spectroscopy are compared to those calculated for the modes with Raman activity. To date, the information regarding the vibrational properties of NbTe_2_ is rather scarce, existing only a few reports on this topic. Erdogan and Kirby presented the first schematic representation of the PDC for the undistorted trigonal phase (1T) of NbTe_2_ ^4^, however, due to the simple approach of using the force constants of 1T-TaS_2_ and symmetry considerations based in an approximate ideal structure, the results disagree with the experimental phonon frequencies found by the same group. Battaglia *et al*.^5^ calculated the PDC for trigonal NbTe_2_ using DFT; however, their PDC showed some branches corresponding to negative frequencies as evidence of dynamical instability caused by the use of an unrealistic crystalline structure. Here, the calculation of the PDC for the actual monoclinic structure of NbTe_2_ were performed by DFT methods as described in methods. Figures 13 and 14 shows the calculated PDC and the corresponding density of states for NbTe_2_, respectively. In the latter, a gap occurs for frequencies between *ca*. 227 cm^−1^ and 243 cm^−1^. It is highlighted the relevance of considering the distortion of the Te planes of the real structure (as opposed to the ideal 1T phase), since it provides structural and dynamical stability to the material. For NbTe_2_, the use of the actual structure with the distorted Te planes is essential to avoid obtaining negative frequencies for the acoustic phonons, that is, for the dynamical stability of the lattice. The number of optical phonons in crystals follows the relation: B_*opt*_ = 3N − 3, where N is the number of atoms in the primitive cell^25^, in this case N = 9, so that B_*opt*_ = 24. Group theory analysis indicates that the decomposition into irreducible representations of the optical modes at the zone center is:

$${B}_{opt}=8{A}_{g}+4{A}_{u}+4{B}_{g}+8{B}_{u}.$$

**Figure 15:**
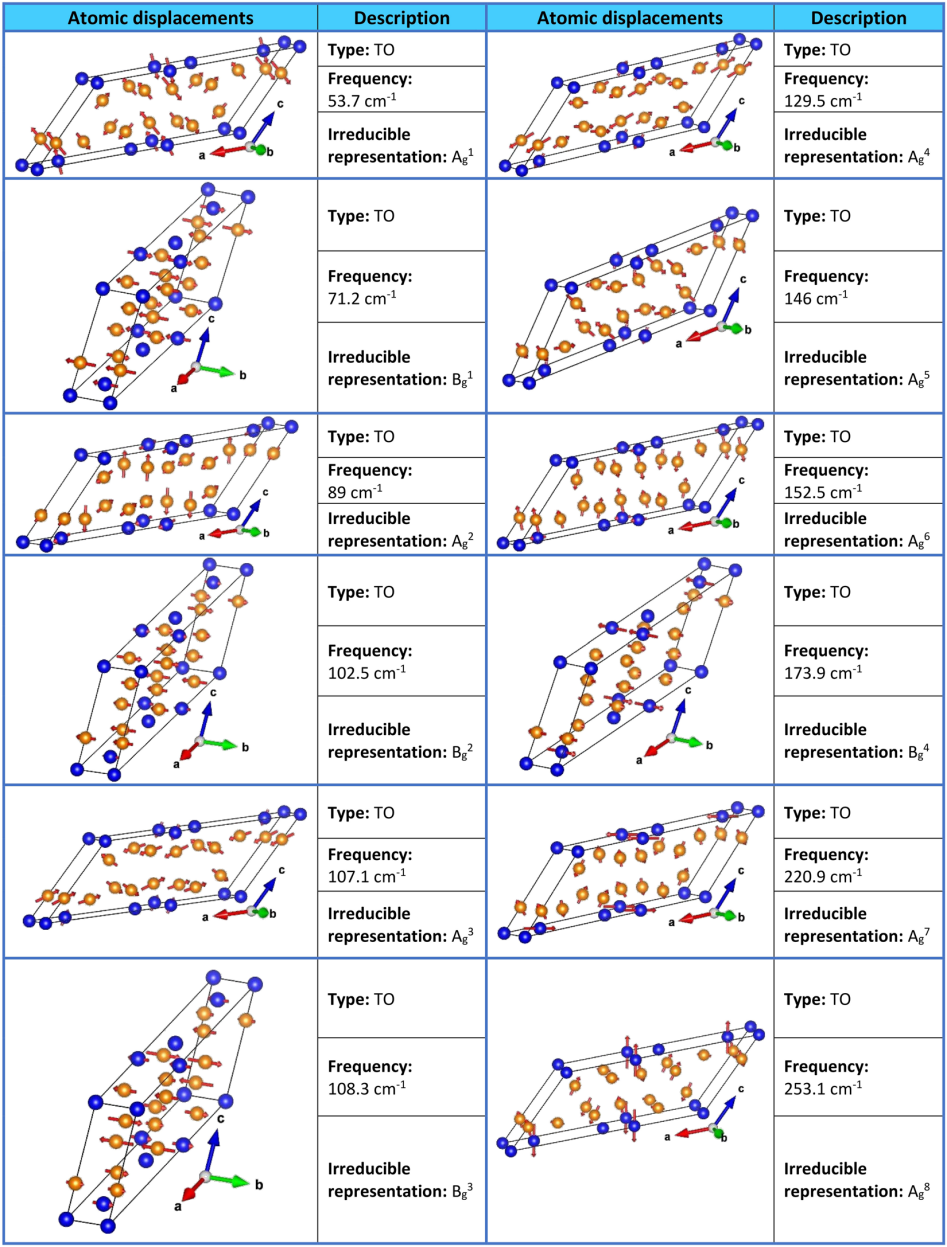
Characteristics of the Raman active normal modes of NbTe_2_.

Twelve modes (8*A*_*g*_ + 4*B*_*g*_) are Raman active, while other twelve (4*A*_*u*_ + 8*B*_*u*_) are active in the infrared. In Fig. 15, the calculated frequencies, type, and irreducible representation of the Raman active normal modes of niobium ditelluride are shown. In addition, a graphic representation for the atomic displacements of each normal mode is included.

**Figure 16:**
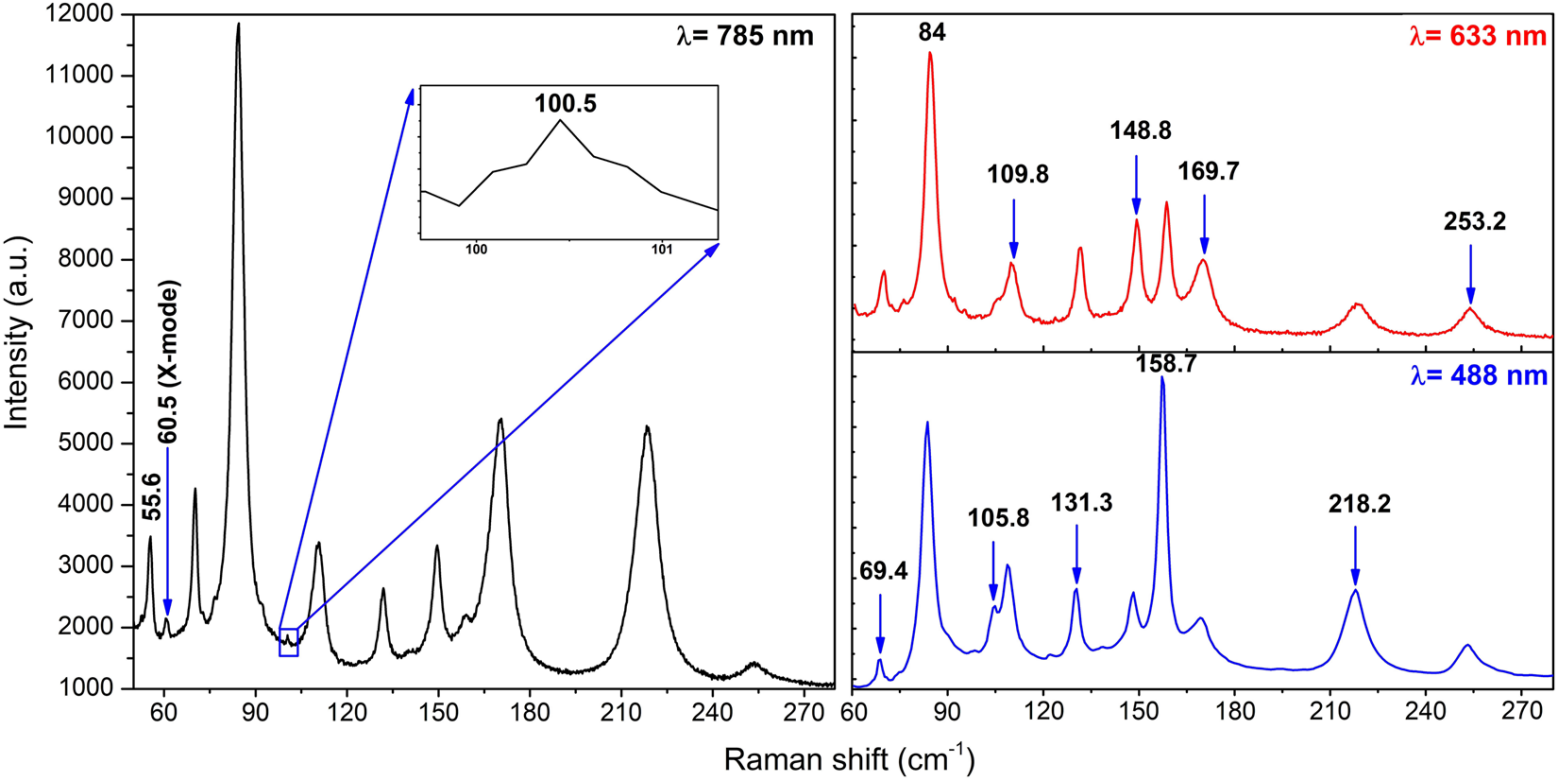
Room temperature Raman spectra of NbTe_2_ acquired with different excitation wavelengths indicated at the topmost right corner of each panel. The inset shows a non-previously reported mode at 100.5 cm^−1^ with B_g_ symmetry.

Erdogan and Kirby^4^ reported the Raman spectrum for NbTe_2_ at different temperatures. They found eleven Raman active modes in the frequency range between 30 cm^−1^ and 260 cm^−1^ using as excitation source a laser with wavelength of 514.5 nm. The calculated frequencies (Fig. 15) indicate that NbTe_2_ has twelve Raman active modes within the range of frequencies measured by Erdogan and Kirby, that is, they could not detect one of these modes (see below). The Raman spectra of NbTe_2_ crystals obtained at room temperature with three different excitation wavelengths: 785 nm, 633 nm and 488 nm are shown in Fig. 16. In this figure the eleven modes reported by Erdogan and Kirby can be observed. It is noticed that the spectra in Fig. 16 present the same peaks, regardless of the excitation wavelength, with different relative intensities. A common feature in the spectra of Fig. 16 is the large intensity of the peak at 84 cm^−1^. This band corresponds to a totally symmetric mode A_g_^2^, Fig. 15. Totally symmetric modes involve usually large variations of the electronic susceptibility, yielding strong peak intensities. The changes in relative intensity of the modes in Fig. 16 for the various excitation energies (1.58 eV/785 nm, 1.96 eV/633 nm and 2.54 eV/488 nm) are a consequence of the different in-resonance or near-resonance electronic transitions promoted for each laser wavelength in the rather intricate electronic band structure of NbTe_2_, Fig. 12, during the inelastic light scattering process. Indeed, the Raman tensor is a function of the resonance or near-resonance inelastic-scattering conditions^26^, which consequently affects the intensity of each vibrational mode since the intensity of a Raman mode is proportional to the change of the Raman tensor components.

**Figure 17:**
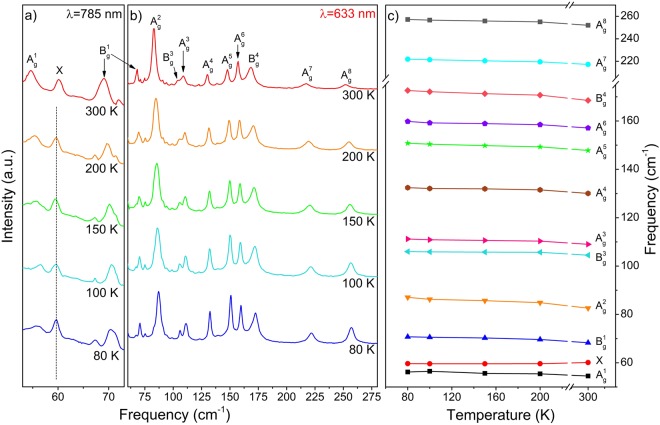
Raman spectra of a NbTe_2_ crystal measured at the indicated temperatures using the excitation lines (**a**) 785 nm, (**b**) 633 nm, (**c**) temperature dependence of the twelve first order (one-phonon) Raman active modes and of the X mode. Note that the frequency of the X mode is temperature independent, while the frequency of the other modes hardens, as the temperature decreases.

Figure 16 shows the eleven Raman bands described in ref.^4^ and two additional peaks. As mentioned above, group theory predicts twelve Raman active modes for NbTe_2_. The calculations indicate a Raman active mode with frequency around 102 cm^−1^, Fig. 15, not reported previously. The inset in Fig. 16 shows the detail of this region where a weak band at 100.5 cm^−1^ can be observed in the spectrum obtained with the 785 nm excitation line. According to our calculations, Fig. 15, this phonon has B_g_ symmetry. Table 2 shows a comparison between the experimental frequencies of the Raman modes measured in this work, those reported in ref.^4^ and the DFT calculated frequencies (Fig. 15). It may be observed that a good agreement exists between the experimental and calculated frequencies. Instrumentally, the acquisition of Raman spectra with the 785 nm line had two advantages with respect to those obtained with the other two laser lines: i) higher resolution (derived from the diffraction grating optics), and (ii) it was possible to detect modes with frequencies closer to the laser line (each excitation line is used in combination with a particular edge filter with specific laser-line-cutoff characteristics). These are the reasons behind the spectrum initiating at slightly lower frequencies for the 785 nm laser line in Fig. 16, with respect to the spectra obtained with the 633 and 488 nm lines. Derived from this capability, it was possible to detect an additional mode at 60.5 cm^−1^, labeled X-mode in the left panel of Fig. 16. In order to determine the origin of this mode, low temperature Raman spectra were obtained in the 80–300 K range. These spectra are shown in Fig. 17. Lower temperatures yield higher phonon frequencies, as a consequence of the thermally induced lattice contraction. This behavior is observed for the Raman active phonons predicted by group theory for NbTe_2_, as shown in Fig. 17c, which contrasts with the comportment of the X mode, whose frequency remained constant for the whole temperature range, Fig. 17a,c. This behavior suggests a phonon-difference process as the origin of this mode. An assignment for such a process must be based on symmetry considerations since the occurrence probability of physical processes is enhanced around high-symmetry points and directions of the reciprocal lattice. The Brillouin zone center, Γ point, has the highest symmetry since it possesses the crystal symmetry. Figure 2 shows other high symmetry points (A, M and Z) in the first Brillouin zone of niobium ditelluride. Taking into consideration the frequency of the X mode (60.5 cm^−1^) and the characteristics of the phonon dispersion curves around the high symmetry points of the Brillouin zone, Fig. 13, it is reasonable to assign this mode to the phonon-difference process B_g_^4^ (169.7 cm^−1^) − B_g_^3^ (109.8 cm^−1^), where we have used the experimental frequencies of the indicated phonons given in Table 2. In fact, it may be observed in Fig. 13 that at those frequencies several branches in the Z-Γ-A-M directions (shadowed regions in Fig. 13) are rather dipersionless (*i.e*. flat), which translates into maxima of the one-phonon density of states, Fig. 14.

**Table 2:** Comparison between the experimental (this work), calculated by DFT (this work), and reported in ref.^4^ frequencies for the Raman active modes of NbTe_2_.

Experimental, this work	Calculated, this work	Experimental, reported in ref.^4^
55.6	53.7 (A_g_^1^)	56
69.4	71.2 (B_g_^1^)	70
84.0	89.0 (A_g_^2^)	84
100.5	102.5 (B_g_^2^)	—
105.8	107.1 (A_g_^3^)	105
109.8	108.3 (B_g_^3^)	110
131.3	129.5 (A_g_^4^)	131
148.8	146.0 (A_g_^5^)	149
158.7	152.5 (A_g_^6^)	158
169.7	173.9 (B_g_^4^)	169
218.2	220.9 (A_g_^7^)	219
253.2	253.1 (A_g_^8^)	254

## Conclusions

Physical properties of niobium NbTe_2_ were studied experimental and theoretically. For the first-principle calculations, the real crystalline structure of NbTe_2_ was considered, as opposed to previous works where approximate, ideal structures were utilized. The electronic band structure, the phonon dispersion curves and their corresponding density of states were computed within the frame of density functional theory. Other calculated properties include cohesive energy (−13.22 eV/formula unit), heat of formation (−1.38 eV/formula unit), bulk modulus (38.5904 GPa) and its derivative (6.025). Experimental studies on the structural, compositional, electronic and vibrational properties were carried out on NbTe_2_ crystals. The lattice parameters obtained through Rietveld refinement of the X-ray diffraction patterns were in good agreement with atomically-resolved transmission electron microscopy images. The characteristics and contrast of the Fourier transform TEM images indicated high crystalline quality of the NbTe_2_ samples. The elemental distribution at the nanoscale and chemical composition were assessed by atom probe tomography. The work function for NbTe_2_ was 5.32 eV, as determined from ultraviolet photoelectron spectroscopy experiments. From Hall effect measurements the resistivity (1.74 × 10^−4^ Ω·cm), free-electron density (5.4 × 10^21^ cm^−3^) and mobility (6.9 cm^2^/Vs) were obtained. These values, along with a pseudogap formed around the Fermi level in the electronic density of states, are consistent with semimetallic characteristics. Notoriously, the electrical resistivity remains nearly constant from 100 K to 400 K, which indicates that electron-phonon scattering does not play a significant role in the charge transport processes for that temperature range. The atomic displacements for the twelve Raman active normal modes predicted by group theory were determined. The calculated frequencies of these modes were in good correspondence with the values found experimentally. It was determined that the frequency of a non-previously detected Raman mode was 100.5 cm^−1^. From low temperature dependent Raman measurements, a vibrational mode with frequency of 60.5 cm^−1^ was assigned to phonon-difference processes involving phonons associated to high-symmetry directions in reciprocal space. Finally, it is pointed out that most of the physical properties determined in this work have not been previously reported.

## Methods

### Experimental details

Niobium ditelluride crystals were grown by the vapor transport method in an evacuated quartz ampoule. X-ray diffraction measurements were carried out in a Rigaku Dmax 2100 diffractometer using a copper target as radiation source (λ = 1.54056 Å). High-resolution transmission electron microscopy (HRTEM) images were obtained in a Jeol JEM-2200FS transmission electron microscope. The elemental composition was determined with the help of laser-assisted Atom Probe Tomography (APT) analyses, which were performed using a local electrode atom probe system (LEAP™ 5000 XS by Cameca Instruments). The APT analyses were performed at a base temperature of 60 K in laser pulsing mode with a wavelength of of 355 nm, ~10 ps pulse length, 200 kHz pulse frequency, and an energy of 40 pJ, and detection rate of 10 ions per 1000 pulses was maintained. The APT specimens were prepared using a dual-beam focused ion beam (FEI Helios Nanolab 600i) following the procedure described in^27^. Scanning electron microscopy (SEM) images were obtained with the help of a JEOL JSM-7401F system. The ultraviolet photoelectron spectroscopy data were obtained in a Thermo Fisher Escalab 250Xi system employing a Helium I line with an energy of 21.22 eV. A −5V bias was applied to the sample in order to shift the spectrum cutoff away from the spectrometer cutoff region. For the room temperature (22 °C) electrical measurements, an Ecopia HMS5000 Hall Effect system was used to determine resistivity, carrier concentration and mobility. The study of the dependence of electrical resistivity as a function of temperature was carried out in a Nanometrics HL5500PC Hall effect system using the Van der Pauw method. The Raman spectra were obtained in a Horiba LabRAM HR Evolution micro-Raman spectrometer using the 488 nm, 633 nm and 785 nm excitation wavelengths, and a diffraction grating of 1800 gr/mm. All the Raman spectra were measured on fresh cleaved surfaces of a NbTe_2_ crystal.

### Theoretical calculations

First principle calculations were carried out using the VASP code^28–31^, where Perdew–Burke–Ernzerhof (PBE) exchange and correlation functional form of the generalized gradient approximation (GGA) was utilized^32,33^. The plane-wave cut off energy was fixed at 425 eV. The energy convergence between two consecutive self-consistent steps was set to 10^−6^ eV. The atomic positions and lattice parameters were optimized, until the total force acting on each atom (Hellman-Feynman forces) was lower than 0.01 eV/Å. The Brillouin zone was analyzed using the Monkhorst–Pack scheme through a 3 × 14 × 7 grid to relax the structure. The grid was increased to 6 × 28 × 14 in the static and density of states (DOS) calculations. The vibrational properties were calculated using the software PHONON^34,35^ with VASP as external *ab initio* program. For this purpose, a supercell of 144 atoms and a 2 × 6 × 4 mesh of *k* space was used. The convergence parameter for two consecutive self-consistent field steps was fixed to 10^−8^ eV. All the structural representations of the crystal lattice were made with VESTA^36^.

## Data Availability

The datasets generated during the current study are available from the corresponding author on reasonable request.

## References

[1] Chhowalla M (2013). The chemistry of two-dimensional layered transition metal dichalcogenide nanosheets. Nat. Chem..

[2] Wang QH, Kalantar-Zadeh K, Kis A, Coleman JN, Strano MS (2012). Electronics and optoelectronics of two-dimensional transition metal dichalcogenides. Nat. Nanotechno..

[3] Nagata S, Abe T, Terashima S, Ishihara Y, Tsutsumi K (1994). Superconductivity in layered-structure compound NbTe_2_. Phys. B.

[4] Erdogan H, Kirby RD (1989). Raman spectrum and lattice dynamics of NbTe_2_. Solid State Commun..

[5] Battaglia, C. *et al*. Fermi-surface-induced lattice distortion in NbTe_2_. *Phys. Rev. B* **72**, 195114-1–195114-10 (2005).

[6] Brown BE (1966). The crystal structures of NbTe_2_ and TaTe_2_. Acta Crystallogr..

[7] Guinebretière René (2007). X-ray Diffraction by Polycrystalline Materials.

[8] Rodríguez-Carvajal J (1993). Recent advances in magnetic structure determination by neutron powder diffraction. Phys. B.

[9] Grillo V, Rotunno E (2013). STEM_CELL: A software tool for electron microscopy: Part I-simulations. Ultramicroscopy.

[10] Grillo V, Rossi F (2013). STEM_CELL: A software tool for electron microscopy. Part 2 analysis of crystalline materials. Ultramicroscopy.

[11] Müller M, Saxey DW, Smith GDW, Gault B (2011). Some aspects of the field evaporation behaviour of GaSb. Ultramicroscopy.

[12] Mancini L (2014). Composition of wide bandgap semiconductor materials and nanostructures measured by atom probe tomography and its dependence on the surface electric field. J. Phys. Chem. C.

[13] Giustino, F. Equilibrum Structures of Materials: Calculation vs. Experiment. In *Materials Modelling using Density Functional Theory: Properties and Predictions*, pp. 66–86 (Oxford University Press, Oxford, 2014).

[14] Zhang H (2009). Enthalpies of formation of magnesium compounds from first-principles calculations. Intermetallics.

[15] Gaskell, D. R. Heat capacity, Enthalpy, Entropy, and the Third Law of Thermodynamics. In *Introduction to the Thermodinamycs of Materials 4th ed*., pp. 125–172 (Taylor & Francis, London, 2009).

[16] Gaskell, D. R. Auxiliary Functions. In *Introduction to the Thermodinamycs of Materials 4th ed*., pp. 97–123 (Taylor & Francis, London, 2009).

[17] Dronskowski, R. Energy, Enthalpy, Entropy and Gibbs Energy. In *Computational Chemistry of Solid State Materials*, pp. 158–160 (WILEY-VCH, Weinheim, 2005).

[18] Tilley, R. J. D. Supplementary Material to Part 4: Physical Properties. In *Understanding Solids: The Science of Materials*, pp. 543–561 (John Wiley & Sons Ltd, Chichester, 2004).

[19] Birch F (1947). Finite elastic strain of cubic crystals. Phys. Rev..

[20] Brixner LH (1962). Preparation and properties of the single crystalline AB_2_-type selenides and tellurides of niobium, tantalum, molybdenum and tungsten. J. Inorg. Nucl. Chem..

[21] Selte K, Kjekshus A (1965). On the magnetic properties of niobium selenides and tellurides. Acta Chem. Scand..

[22] Derry Gregory N., Kern Megan E., Worth Eli H. (2015). Recommended values of clean metal surface work functions. Journal of Vacuum Science & Technology A: Vacuum, Surfaces, and Films.

[23] Kumar A, Ahluwalia PK (2013). Effect of quantum confinement on electronic and dielectric properties of niobium dichalcogenides NbX_2_ (X = S, Se, Te). J. Alloys Compd..

[24] Burns, G. *Solid State Physics*, p. 339 (Academic Press, San Diego, 1985).

[25] Srivastava, G. P. *The Physics of Phonons*, p. 32 (Adam Hilger, Bristol, 1990).

[26] Snoke, D. W. *Solid State Physics: Essential Concepts*, p. 367 (Addison-Wesley, San Francisco, 2009).

[27] Thompson K (2007). *In-situ* site‐specific specimen preparation for atom probe tomography. Ultramicroscopy.

[28] Kresse G, Hafner J (1993). Ab initio molecular dynamics for liquid metals. Phys. Rev. B.

[29] Kresse G, Hafner J (1994). Ab initio molecular-dynamics simulation of the liquid-metal-amorphous-semiconductor transition in germanium. Phys. Rev. B.

[30] Kresse G, Furthmüller J (1996). Efficiency of ab-initio total energy calculations for metals and semiconductors using a plane-wave basis set. Comput. Mater. Sci..

[31] Kresse G, Furthmüller J (1996). Efficient iterative schemes for ab initio total-energy calculations using a plane-wave basis set. Phys. Rev. B.

[32] Perdew JP, Burke K, Ernzerhof M (1996). Generalized gradient approximation made simple. Phys. Rev. Lett..

[33] Perdew JP, Burke K, Ernzerhof M (1997). Erratum: Generalized gradient approximation made simple. Phys. Rev. Lett..

[34] Parlinsky, K. *Software PHONON*, Cracow (2013).

[35] Parlinski K, Li Z, Kawazoe Y (1997). First-Principles determination of the soft mode in cubic ZrO_2_. Phys. Rev. Lett..

[36] Momma K, Izumi F (2011). VESTA 3 for three-dimensional visualization of crystal, volumetric and morphology data. J. Appl. Crystallogr..

